# Cerebral autoregulation in orthostatic hypotension and falls among older adults: a community-based exploratory study

**DOI:** 10.1007/s10286-025-01152-6

**Published:** 2025-09-08

**Authors:** Nor Izzati Saedon, James Frith, Wan Azman Wan Ahmad, Maw Pin Tan

**Affiliations:** 1 Faculty of Medicine, Department of Medicine, Ageing and Age-Associated Disorders Research Group, Division of Geriatric Medicine, University of Malaya, 50603 Kuala Lumpur, Malaysia; 2 Consultant of Geriatric Medicine, Falls and Syncope Service, Older People’s Medicine, The Campus of Ageing and Vitality, Newcastle upon Tyne, UK; 3 Division Cardiology, Senior Consultant Cardiologist, University Malaya Medical Centre, 59100 Kuala Lumpur, Malaysia

**Keywords:** Orthostatic hypotension, Cerebral autoregulation, Falls, Older adults, Cerebral blood flow

## Abstract

**Background:**

Orthostatic hypotension (OH) is prevalent in older adults and is often associated with falls. However, the presence or absence of symptoms in OH may be mediated by cerebral autoregulation, which helps maintain cerebral perfusion during blood pressure fluctuations.

**Methods:**

We recruited 40 older adults (aged ≥ 55 years) from the Malaysian Elders Longitudinal Research (MELoR) cohort. Participants underwent cerebral blood flow velocity monitoring using transcranial Doppler ultrasonography and beat-to-beat blood pressure recording. Three protocols were used: active stand, mental arithmetic, and Valsalva manoeuvre. Participants were categorized, based on OH (≥ 30 mmHg systolic drop) and fall history, into four groups. Cerebrovascular resistance (CVR) was derived and analysed.

**Results:**

Participants with OH but no history of falls demonstrated preserved autoregulatory responses, as reflected by adaptive reductions in CVR. In contrast, fallers—regardless of OH status—had impaired CVR modulation. Significant group differences were found during the active stand test at 165 s and 180 s (*p* < 0.05).

**Conclusion:**

Preserved cerebral autoregulation may protect older adults with OH from symptomatic manifestations such as falls. Targeting cerebral autoregulation could offer novel approaches for preventing falls in this population.

## Introduction

Orthostatic hypotension (OH) is a common and under-recognized clinical condition in older adults, defined as a sustained drop in systolic blood pressure (SBP) of ≥ 20 mmHg or diastolic blood pressure (DBP) of ≥ 10 mmHg upon standing, or ≥ 30 mmHg SBP in some older cohorts [1]. It affects up to 30% of community-dwelling older individuals and is associated with adverse outcomes including falls, fractures, cognitive impairment, and increased mortality [2–4]. With a rapidly aging global population, understanding the variability in clinical expression among those with OH is essential for targeted interventions.

Although OH is typically diagnosed via peripheral haemodynamic measurements, emerging evidence suggests that central mechanisms—particularly cerebral autoregulation—play a critical role in modulating symptom manifestation [5, 6]. Cerebral autoregulation refers to the brain’s intrinsic ability to maintain stable cerebral blood flow (CBF) despite fluctuations in systemic blood pressure within a defined range [7]. This mechanism safeguards cerebral perfusion during physiological stressors, ensuring adequate oxygen and nutrient delivery to brain tissue, which is essential for cognitive and motor function [8]. Age-related changes, as well as comorbid conditions such as diabetes, hypertension, and neurodegenerative diseases, can impair autoregulatory efficiency [9, 10]. Consequently, some older adults with OH may experience symptoms like dizziness or falls due to inadequate cerebral perfusion, while others remain asymptomatic—suggesting a buffering role of preserved autoregulation [11].

Previous studies have investigated cerebral autoregulation in selected clinical populations. For instance, Novak et al. demonstrated variability in cerebral blood flow responses among patients with OH with autonomic failure [1], while Mankovsky et al. reported impaired autoregulation in diabetic patients with autonomic neuropathy [2]. However, these studies were largely hospital-based, limiting their generalizability. To address this gap, we assess cerebral autoregulation among community-dwelling older adults with and without OH, stratified by fall history. We hypothesized that preserved autoregulatory function would differentiate asymptomatic from symptomatic OH, and that impaired cerebrovascular response may contribute to fall risk independently of OH.

## Materials and methods

### Study design and setting

This was a cross-sectional, observational study conducted at the University of Malaya Medical Centre in Kuala Lumpur, Malaysia. Ethical approval was obtained from the institutional review board (ethics number), and all participants provided written informed consent prior to participation. The study was conducted in accordance with the principles of the Declaration of Helsinki.

### Participants and recruitment

Participants were drawn from the Malaysian Elders Longitudinal Research (MELoR) cohort, a community-based longitudinal study focusing on aging and functional health in older adults. The MELoR cohort includes community-dwelling adults aged ≥ 55 years. Participants were recruited based on willingness and ability to complete neurocardiovascular assessments. Additional participants were recruited through outpatient clinics and local advertisements. Inclusion criteria included age ≥ 55 years, ability to provide informed consent, and physical capacity to undergo protocol assessments (total number screened *n* = 64; final *n* = 40). Exclusion criteria were the presence of known focal neurological deficits, prior history of significant carotid stenosis, or the inability to obtain a suitable transtemporal window for transcranial Doppler (TCD) measurement.

### Clinical assessment and grouping detailed

Clinical interviews were conducted to document demographics, comorbidities, medication use, and history of falls within the previous 12 months. Falls were defined as any unexpected event resulting in the individual coming to rest on the ground, floor, or lower level. Orthostatic hypotension was diagnosed based on a systolic blood pressure reduction of ≥ 30 mmHg during the active stand test. Participants were categorized into four groups:

- Group 1: No OH, no falls
- Group 2: OH, no falls
- Group 3: No OH, with falls
- Group 4: OH, with falls

### Haemodynamic monitoring protocols

All participants fasted for 10 h before assessments and were instructed to continue taking prescribed medications. Continuous beat-to-beat blood pressure was monitored using a TaskForce^®^ Monitor (CNSystems, Austria), utilizing the vascular unloading technique. The device was calibrated using oscillometric brachial blood pressure measurements.

Cerebral blood flow velocity (CBFV) was assessed using a 2-MHz pulsed TCD probe (DWL DopplerBox^®^, Germany) to insonate the left middle cerebral artery via a transtemporal window. CBFV signals were synchronized with blood pressure and electrocardiogram (ECG) data.

Cerebrovascular resistance calculation: Cerebrovascular resistance (CVR) was derived from mean arterial pressure (MAP) and CBFV using the formula: CVR = MAP/CBFV. intracranial pressure was assumed to be stable and negligible across all participants.

### Experimental challenge protocols

Three physiological challenge protocols were performed:

1. Active stand test: After 5 min supine rest, participants stood upright within 3 s and remained standing for 3 min. CBFV and blood pressure data were collected throughout.
2. Mental arithmetic test: Participants performed continuous serial subtractions from 100 in intervals of seven for 1 min while seated.
3. Valsalva manoeuvre: Participants blew into a manometer to maintain 40 mmHg pressure for 15 s. Measurements were analysed across the four standard phases of the Valsalva response.

### Data analysis

CBFV and blood pressure readings were extracted at predefined time points. MATLAB (MathWorks, USA) and SPSS (Version 26.0, IBM) were used for signal processing and statistical analysis. Analysis of variance (ANOVA) and linear regression were used to identify group differences and adjust for potential confounders such as age, sex, body mass index (BMI), and antihypertensive use. A one-way ANOVA was used for group comparisons, and Tukey’s honestly significant difference (HSD) post hoc test was employed for pairwise analysis where appropriate.

## Results

**Fig. 1:**
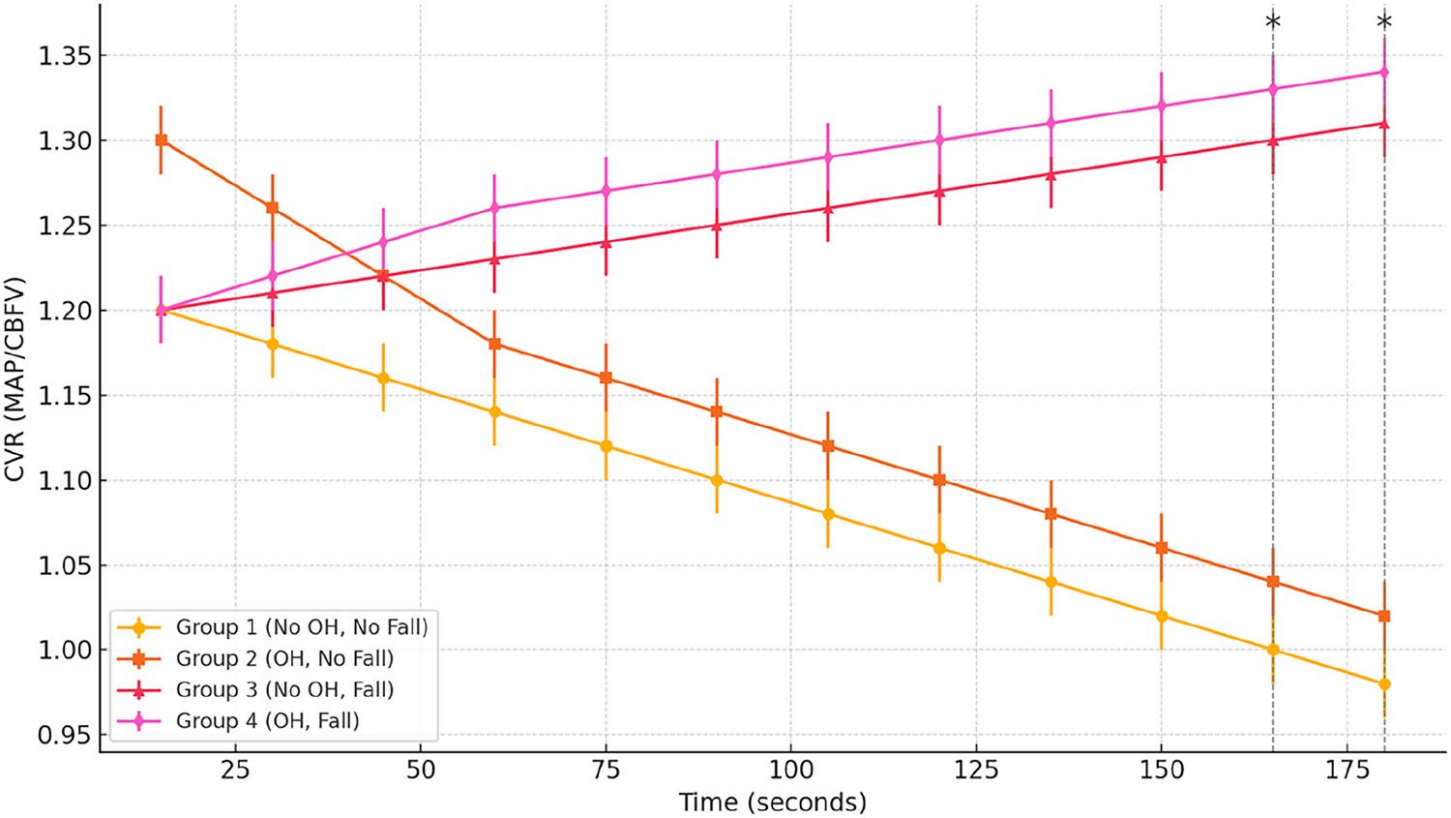
Mean cerebrovascular resistance (CVR) over 180 s during active stand test. This figure illustrates group-wise CVR changes from 15 to 180 s during the active stand protocol. Groups 1 and 2 showed decreasing trends, while Groups 3 and 4 exhibited blunted or upward trends, indicating impaired autoregulation in fallers

A total of 40 participants were included in the final analysis. The distribution across the four groups was as follows: Group 1 (No OH, no fall), 22 participants (55%); Group 2 (OH, no fall), eight participants (20%); Group 3 (No OH, fall), six participants (15%); and Group 4 (OH, fall), four participants (10%). Baseline demographic and clinical characteristics are summarized in Table 1. No statistically significant differences were found in age, gender, BMI, or cognitive scores across the groups. However, use of anti-Parkinsonian medications was more common in the faller groups (*p* = 0.040). Haemodynamic responses recorded during the active stand, mental arithmetic, and Valsalva manoeuvre protocols are presented in Table 2. During active stand, a significant difference was observed in standing systolic blood pressure between groups (*p* = 0.036), with Group 2 and Group 4 displaying lower values than non-OH participants. Cerebrovascular resistance trends in Fig. 1 illustrate the mean CVR values across the 180-s active stand protocol. While Groups 1 and 2 demonstrated a relatively stable or slightly decreasing trend in CVR over time, Groups 3 and 4 exhibited minimal or no adaptive changes, indicating impaired autoregulatory capacity.

**Table 1:** Participant characteristics by group (*n* = 40)

Characteristic	Group 1 (No OH, no fall) (*n* = 22)	Group 2 (OH, no fall) (*n* = 8)	Group 3 (No OH, fall) (*n* = 6)	Group 4 (OH, fall) (*n* = 4)	*p*-value
Number of participants	22	8	6	4	–
Age (years), mean (SD)	68.1 (6.3)	70.4 (5.9)	69.2 (7.2)	71.0 (6.1)	0.472
Female, *n* (%)	13 (59%)	5 (63%)	3 (50%)	2 (50%)	0.904
BMI (kg/m^2^), mean (SD)	25.4 (3.8)	24.7 (4.2)	25.1 (3.5)	24.9 (3.9)	0.893
MMSE score, mean (SD)	26.7 (2.1)	26.1 (2.5)	26.4 (2.3)	25.9 (2.6)	0.833
MoCA score, mean (SD)	23.5 (2.8)	22.9 (2.9)	23.2 (2.6)	22.8 (2.7)	0.751
Antihypertensive use, *n* (%)	11 (50%)	5 (63%)	3 (50%)	3 (75%)	0.802
Anti-Parkinsonian medications, *n* (%)	0 (0%)	0 (0%)	2 (33%)	1 (25%)	0.040 *

*BMI* body mass index, *MMSE* Mini-Mental State Examination, *MoCA* Montreal Cognitive Assessment

*Statistically significant at *p* < 0.005

**Table 2:** Haemodynamic measures across challenge protocols

Measure	Group 1 (No OH, no fall)	Group 2 (OH, no fall)	Group 3 No OH, fall	Group 4 (OH, fall)	*p*-value
Supine SBP (mmHg)	142.6 (± 19.5)	140.2 (± 16.1)	145.0 (± 21.3)	138.8 (± 15.9)	0.822
Standing SBP (mmHg)	138.1 (± 21.2)	121.3 (± 18.7)	137.7 (± 19.4)	118.6 (± 16.5)	0.036 *
SBP drop (mmHg)	4.5 (± 5.1)	18.9 (± 3.4)	5.2 (± 4.9)	20.2 (± 5.1)	< 0.001 *
Supine DBP (mmHg)	76.2 (± 9.1)	74.5 (± 10.0)	75.8 (± 8.3)	74.0 (± 7.9)	0.921
Standing DBP (mmHg)	72.5 (± 10.5)	66.7 (± 11.8)	70.9 (± 10.0)	65.4 (± 9.3)	0.188
HR supine (bpm)	69.8 (± 8.7)	70.4 (± 9.2)	68.6 (± 8.0)	71.3 (± 7.9)	0.934
HR standing (bpm)	76.5 (± 10.2)	77.9 (± 10.4)	75.1 (± 9.6)	78.4 (± 9.1)	0.879

Note: *SBP*   systolic blood pressure, *DBP* diastolic blood pressure, *HR*  heart rate. Values shown are mean (± SD)

*Statistically significant at *p* < 0.05

**Fig. 2:**
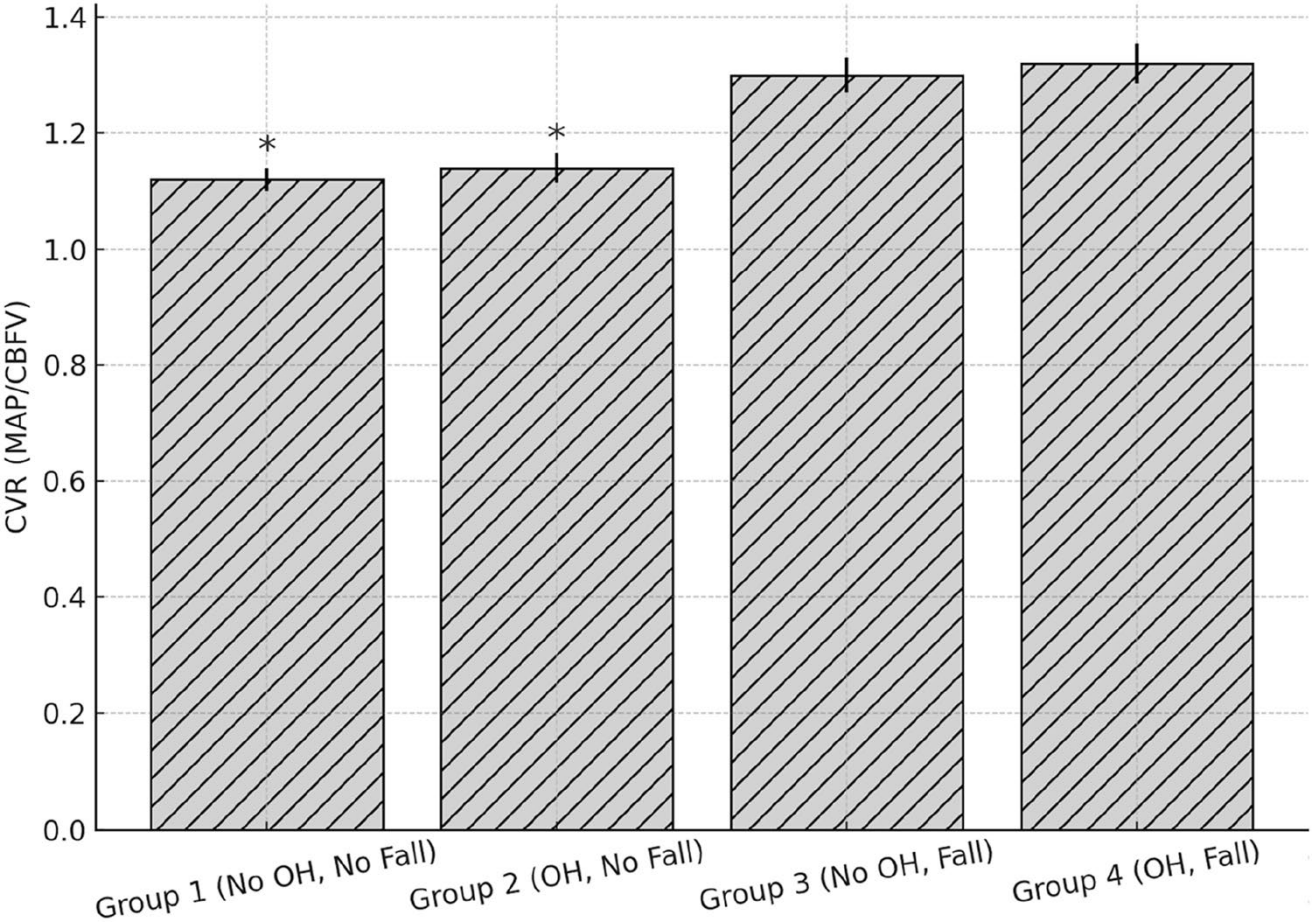
CVR response during mental arithmetic by group. This figure shows the cerebrovascular resistance (CVR) response during the 1-min mental arithmetic test. Groups 1 and 2 demonstrated appropriate increases in CVR, whereas Groups 3 and 4 showed flat or erratic responses, suggesting reduced neurovascular responsiveness

Significant group differences in CVR were observed at 165-s (*p* = 0.028) and 180-s (*p* = 0.023) time points during the active stand. These findings are detailed in Supplementary Table 4. Notably, Group 3 (No OH, fall) showed the greatest CVR reduction failure compared to Group 1. Mental arithmetic test and Valsalva manoeuvre: During the mental arithmetic test, CVR increased consistently in Group 1 and Group 2, reflecting intact cerebrovascular responsiveness to cognitive load. This pattern was disrupted in both faller groups, which exhibited blunted or inconsistent CVR modulation (Fig. 2).

**Fig. 3:**
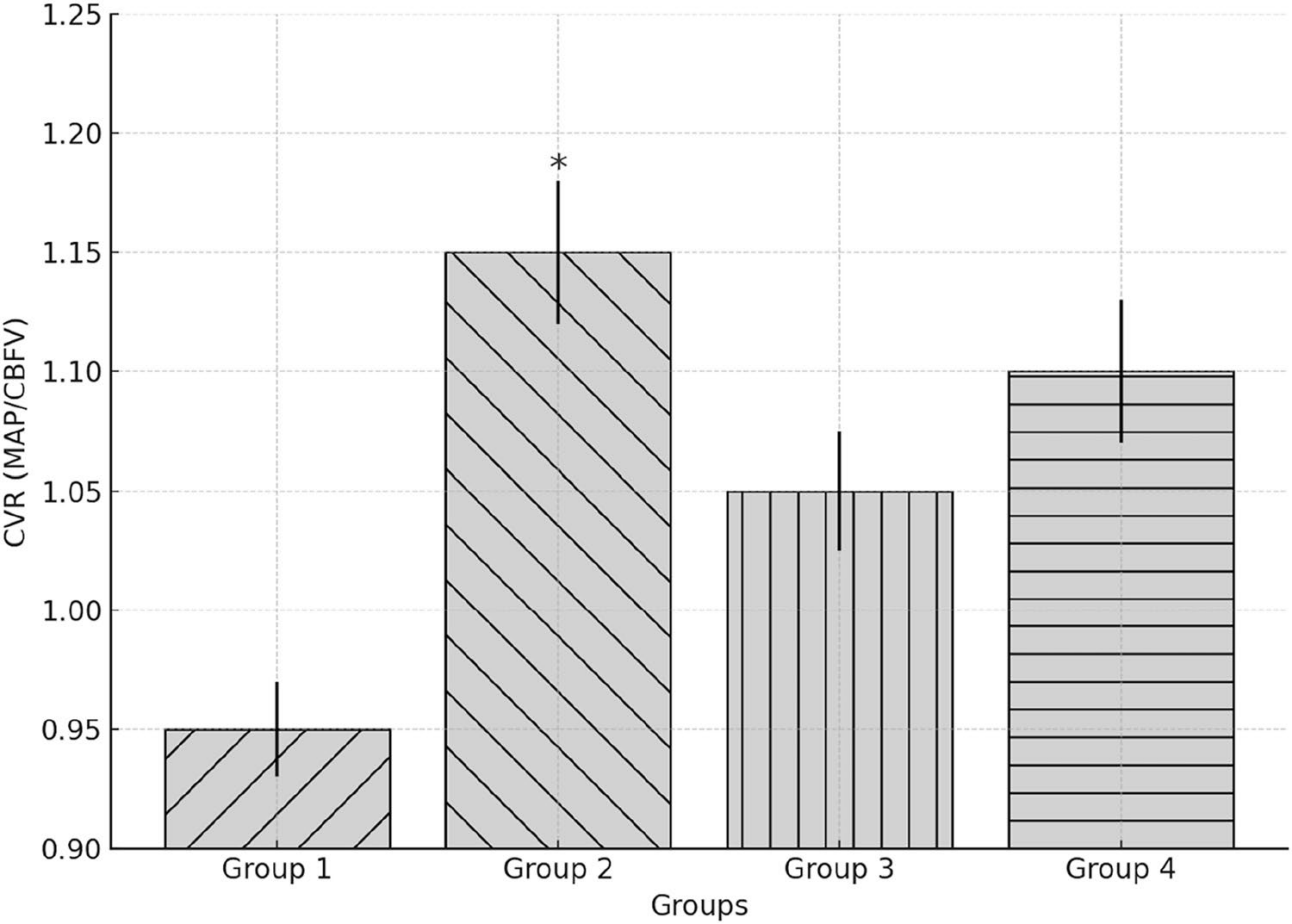
CVR response during valsalva manoeuvre by group. This figure shows CVR patterns throughout the Valsalva manoeuvre. A paradoxical increase during phase 2B was observed in Group 2, contrasting with expected trends in Group 1. Blunted or abnormal patterns were evident in faller groups (Groups 3 and 4)

With the Valsalva manoeuvre (Fig. 3), Group 2 exhibited a paradoxical rise in CVR during phase 2B, contrasting with the expected drop seen in Group 1. This may indicate an enhanced baroreceptor-mediated vasoconstrictive response to counter intrathoracic pressure changes. Mean differences during each phase are reported in supplementary Table 1.3. Analysis of a multivariable regression model adjusting for age, standing systolic BP, and Parkinson’s medication use revealed that Group 2 had significantly lower CVR at 180 s than Group 1 (β = −0.213, 95% CI 0.057–0.798, *p* < 0.05). Group 3 and 4 differences were not statistically significant after adjustment (Table 3).

**Table 3:** Adjusted multivariate regression for CVR at 180 s during active stand

Comparison group vs Group 1	Beta coefficient	95% Confidence interval	*p*-value
Group 2 (OH, no fall)	−0.213	0.057–0.798	< 0.05*
Group 3 (No OH, fall)	−0.056	−0.128 to 0.079	0.423
Group 4 (OH, fall)	−0.034	−0.099 to 0.092	0.671

Adjusted for age, standing systolic blood pressure, and Parkinson’s medication use

*Statistically significant at *p* < 0.005

## Discussion

This study provides new insights into the potential role of cerebral autoregulation in explaining why some older adults with orthostatic hypotension (OH) remain asymptomatic while others develop falls. Our results demonstrate that participants with OH but no history of falls (Group 2) exhibited preserved cerebrovascular resistance (CVR) modulation across physiological challenges, suggesting intact autoregulatory mechanisms. In contrast, fallers—both with and without OH (Groups 3 and 4)—had impaired CVR responses, supporting the hypothesis that cerebral autoregulation serves as a protective buffer against orthostatic symptoms.

This interpretation is consistent with Novak et al. [1], who found that patients with autonomic failure and OH could remain asymptomatic due to preserved cerebral autoregulation. Mankovsky et al. [2] also reported impaired autoregulation in diabetic patients with OH, linking dysfunction to symptomatic presentations. Unlike previous hospital-based studies, our community-based approach using the Malaysian Elders Longitudinal Research (MELoR) cohort increases the generalizability of our findings. However, it should be noted that inclusion was based on participants’ ability to complete assessments, and not all fallers within the cohort were screened—this limits the ability to determine the true prevalence of autoregulatory impairment in the wider population.

Interestingly, the paradoxical rise in CVR observed during phase 2B of the Valsalva manoeuvre in Group 2 may reflect an augmented baroreceptor-mediated vasoconstrictive response, suggesting possible compensatory autonomic adaptations. The inclusion of this manoeuvre allowed for deeper exploration of cardiovagal responses. Nevertheless, further evaluation of heart rate variability and baroreflex sensitivity would strengthen our understanding of the underlying autonomic mechanisms. A study conducted among individuals with symptomatic and asymptomatic carotid sinus hypersensitivity (CSH) had previously demonstrated similar findings in that individuals with asymptomatic (CSH) appeared to have enhanced cerebrovascular responses to hypotensive challenge compared to individuals without falls or CSH [12]. This previous study had not included fallers without CSH. As both CSH and OH are hypotensive disorders affecting primarily older adults, this study, therefore, confirms previous suspicion that impaired cerebral autoregulation may also be present in individuals without OH with a history a fall. This does not, however, necessarily imply that OH then may not have a role in fall occurrence, since active stand was only performed on one occasion, with previous studies indicated that lying and standing blood pressure tests may need to be repeated to confirm the diagnosis of OH [13]. In addition, co-occurrence of OH with vasovagal syncope or CSH has also been documented [14].

Since falls tend to be multifactorial, even with the absence of OH, the older adult may still have multiple medical conditions which may negatively affect cerebral autoregulation [15]. This is supported by Finucane et al. [8], who showed that cerebral perfusion measures may better explain fall risk than orthostatic blood pressure measurements alone. Common conditions culminating in increased falls risk which may negatively affect cerebral autoregulatory capacity include cerebrovascular disease, gait disorders, neurocognitive disorders [16] and head trauma [17].

Transcranial Doppler (TCD) ultrasonography measures cerebral blood flow velocity rather than absolute flow and hence is reliant on the assumption that intracranial pressure (ICP) remains stable [18]. While we excluded participants with known neurological pathology such as normal-pressure hydrocephalus (NPH), we acknowledge that silent or subclinical ICP variations could influence results. TCD remains a validated tool for autoregulation studies, but its limitations—such as operator dependency and poor acoustic windows in some individuals—must be recognized [19].

The small sample size, especially in Group 4 (OH with falls), introduces concerns about statistical power and group comparability. Future studies should include larger, balanced samples and possibly explore delayed OH by extending the active stand duration beyond 3 min [20]. Moreover, individual variability in blood pressure thresholds for cerebral hypoperfusion and falls should be explored longitudinally. Not all OH is immediately symptomatic; asymptomatic cases may reflect early compensatory phases prior to autoregulatory decompensation [21].

Lastly, compensatory heart rate (HR) responses were assessed, but no statistically significant group differences emerged. These findings suggest a dissociation between HR and CVR in this context, but further autonomic profiling may elucidate differential cardiovagal contributions to symptom manifestation.

### Limitations

Several limitations should be noted. First, the sample size in Group 4 (OH with falls) was small (*n* = 4), which limits the statistical power and generalizability of findings involving this subgroup. Comparisons involving this group should be interpreted with caution. However, given the exploratory nature of the study, the observed trends remain valuable for hypothesis generation and informing future larger-scale research. Second, cerebral blood flow was measured using transcranial Doppler ultrasonography, which estimates flow velocity rather than absolute perfusion. Although this is a validated surrogate for cerebral autoregulatory assessment, it does not capture volumetric blood flow. Third, the cross-sectional design prevents inference of causal relationships. Longitudinal studies would be required to determine whether impaired autoregulation predicts future falls.

Lastly, potential confounding factors such as sleep quality, frailty status, and unmeasured comorbidities could influence cerebrovascular responses. These factors should be explored in future studies.

## Conclusion

Our findings suggest that preserved cerebral autoregulation differentiates asymptomatic OH from symptomatic OH associated with falls. This autoregulatory integrity may play a protective role in older adults, mitigating the cerebral hypoperfusion effects of blood pressure fluctuations. By integrating cerebrovascular resistance assessments into clinical evaluations, healthcare providers can better identify individuals at elevated fall risk, even in the absence of overt orthostatic hypotension (OH). Future research should further explore interventions that support or enhance autoregulatory function to reduce morbidity among older populations.

## Data Availability

The Malaysian Elders Longitudinal Research (MELoR) dataset is not publicly available due to privacy and ethical restrictions, but deidentified data may be shared in accordance with institutional and ethics committee guidelines.
